# *Drosophila* Graf regulates mushroom body β-axon extension and olfactory long-term memory

**DOI:** 10.1186/s13041-021-00782-x

**Published:** 2021-04-23

**Authors:** Sungdae Kim, Joohyung Kim, Sunyoung Park, Joong-Jean Park, Seungbok Lee

**Affiliations:** 1 Department of Cell and Developmental Biology and Dental Research Institute, Seoul National University, Seoul, 08826 Republic of Korea; 2 Department of Brain and Cognitive Sciences, Seoul National University, Seoul, 08826 Republic of Korea; 3 Department of Physiology, College of Medicine, Korea University, Seoul, 02841 Republic of Korea

**Keywords:** *Drosophila*, Intellectual disability, Graf/oligophrenin-1, EGFR signaling, Mushroom body development

## Abstract

Loss-of-function mutations in the human *oligophrenin-1* (*OPHN1*) gene cause intellectual disability, a prevailing neurodevelopmental condition. However, the role OPHN1 plays during neuronal development is not well understood. We investigated the role of the *Drosophila* OPHN1 ortholog Graf in the development of the mushroom body (MB), a key brain structure for learning and memory in insects. We show that loss of Graf causes abnormal crossing of the MB β lobe over the brain midline during metamorphosis. This defect in *Graf* mutants is rescued by MB-specific expression of Graf and OPHN1. Furthermore, MB α/β neuron-specific RNA interference experiments and mosaic analyses indicate that Graf acts via a cell-autonomous mechanism. Consistent with the negative regulation of epidermal growth factor receptor (EGFR)-mitogen-activated protein kinase (MAPK) signaling by Graf, activation of this pathway is required for the β-lobe midline-crossing phenotype of *Graf* mutants. Finally, *Graf* mutants have impaired olfactory long-term memory. Our findings reveal a role for Graf in MB axon development and suggest potential neurodevelopmental functions of human OPHN1.

## Introduction

Intellectual disability (ID) is a neurodevelopmental disorder defined by significant impairments in both intellectual functioning and adaptive behavior that affects approximately 1% of the global population [1]. The underlying causes of ID are highly heterogeneous, including environmental factors and/or genetic changes affecting ~ 1000 genes [2]. The human *oligophrenin-1* (*OPHN1*) gene was first associated with ID by molecular analysis of an X;12 balanced translocation in a patient [3, 4]. Subsequently, several loss-of-function mutations in *OPHN1* have been found in families with syndromic ID associated with cerebellar hypoplasia and, in some cases, with ventricular dilation [5–8]. *Ophn1* deficiency in mice recapitulates some of the human pathologies including behavioral and cognitive abnormalities, as well as ventricular dilation [9], supporting the notion that loss of *OPNH1* function is responsible for syndromic ID.

The OPHN1 protein belongs to the Graf (GTPase regulator associated with focal adhesion kinase-1) subfamily of GTPase-activating proteins (GAPs), whose members commonly comprise an N-terminal Bin/amphiphysin/Rvs (BAR) domain, a pleckstrin homology (PH) domain, and a RhoGAP domain [10]. Whereas other Graf subfamily members (GRAF1, GRAF2, and GRAF3) contain a C-terminal Src homology 3 (SH3) domain, OPHN1 possesses a proline-rich domain. The BAR and PH modules of OPNH1 produce or sense membrane curvature [11], and the GAP domain inhibits Rho-family small GTPases, the master regulators of actin dynamics [3, 12, 13]. The proline-rich domain of OPHN1 interacts with the endocytic protein endophilin [14, 15], suggesting that OPHN1 may regulate endocytosis by orchestrating actin and membrane dynamics. OPHN1 is highly expressed in the brain and localizes to neuronal pre- and postsynaptic compartments [13]. Postsynaptic OPHN1 regulates synaptic structure, function, and plasticity by controlling α-amino-3-hydroxy-methylisoazol-4-propionate (AMPA) receptor internalization and/or stability [9, 13, 16, 17], whereas presynaptic OPHN1 is required for efficient synaptic vesicle endocytosis [15, 16]. Both of these functions appear to involve OPHN1′s GAP activity toward RhoA and interactions with endophilin A1 [13, 15–17]. Precise regulation of Rho-GTPase signaling is also important for neurite outgrowth and axon pathfinding during neuronal development [18]. However, it is not known whether the RhoGAP protein OPHN1 plays additional roles in the developing nervous system.

The *Drosophila* genome contains a single ortholog of the human *Graf* gene family (*Graf*). The *Drosophila* Graf protein has an identical domain organization (except its C-terminal SH3 domain) and displays 33% identity and 50% similarity to human OPHN1 [19]. In this study, we characterized the role of Graf in the developing mushroom body (MB), which is a key brain structure for olfactory learning and memory in insects [20] and is an excellent model for the study of gene functions in neuronal development. We show that Graf is required for MB β-lobe axons to stop at the brain midline. Expression pattern and mosaic analyses demonstrate that Graf acts via a cell-autonomous mechanism, and genetic interaction experiments suggest that Graf regulates β-lobe axon extension by downregulating the epidermal growth factor receptor (EGFR)-mitogen-activated protein kinase (EGFR-MAPK) pathway. Finally, we show that Graf is required for the formation of olfactory long-term memory. Altogether, our results define a role for Graf in establishing neuronal wiring patterns and may contribute to a better understanding of the pathogenesis of *OPHN1*-induced ID.

## Materials and methods

### *Drosophila* strains

Flies were maintained on standard *Drosophila* yeast-cornmeal molasses food at 25 °C. The wild-type strain used in this study was *w*^*1118*^. The *Graf* null mutant, *Graf*^*1*^, and transgenic *UAS-Graf-HA* flies were described previously [19]. Transgenic lines carrying *Graf-GAL4* and *UAS-OPHN1-HA* were generated in the *w*^*1118*^ background by standard procedures. The following fly strains were obtained from the Bloomington Stock Center (Bloomington, IN, USA): *Df(1)BSC756* (a deficiency of the *Graf* locus), *UAS-Graf*^*RNAi*^*, Egfr*^*f24*^, *rl*^*1*^, *UAS-mCD8-GFP*, *UAS-NLS-mCherry*, and *UAS-EGFR*^*CA*^. *rl*^*10*^ was obtained from Ernst Hafen (ETH Zurich, Switzerland). The following GAL4 lines were used as drivers of upstream activation sequence (UAS) transgenes: *C155-GAL4* [21], *OK107-GAL4* [22], c*739-GAL4*, *c305a-GAL4*, and *1471-GAL4* [23]. The following fly strains for mosaic analysis with a repressible cell marker (MARCM) of MB neurons were obtained from the Bloomington Stock Center: *FRT19A* and *hs-FLP*,*tubP-GAL80*,*FRT19A*/*Y*; *UAS-mCD8-GFP*/*CyO*; *OK107-GAL4*.

### MARCM-based clonal analysis

To generate mitotic single cell clones in the MB, we used the MARCM technique as described previously [24]. To produce green fluorescent protein (GFP)-labeled wild-type and *Graf* mutant clones, *hs-FLP*,*tubP-GAL80*,*FRT19A*/*Y*; *UAS-mCD8-GFP*/*CyO*; *OK107-GAL4* flies were crossed to either *FRT19A* or *Graf*^*1*^,*FRT19A* flies. The MARCM-ready animals were subjected to heat shock (37 °C for 40 min) at the pupal stage. Female adult brains were stained with anti-GFP and anti-FasII antibodies to label MARCM clones and the α/β-lobe area. Fifteen brains were examined for marked MB clones.

### Molecular biology

For generation of a *Graf-GAL4* driver, a 4697 bp fragment of the *Graf* 5′ region (− 4967 to + 1 relative to the translation starting site) was PCR amplified from the genomic clone BACR23C18 (Children’s Hospital Oakland Research Institute, Oakland, CA, USA) and inserted into the pCaspeR4 vector (Addgene, Watertown, MA, USA). Subsequently, the *GAL4* gene and the *Drosophila* hsp70 terminator were excised from the pGaTB vector [25] and subcloned into the 3′ end of the *Graf* promoter.

For transgenic rescue experiments, the full-length cDNA of human *OPHN1* was amplified by reverse transcription PCR (RT-PCR) of total RNA prepared from HeLa cells and then inserted into pGEM-T Easy vector (Promega, Madison, WI, USA). The human *OPHN1* cDNA insert was subcloned into the pcDNA3.1-HA vector, a derivative of the pcDNA3.1( +) vector (Invitrogen, Carlsbad, CA, USA), and then moved with the corresponding hemagglutinin (HA) tag into the pUAST vector to produce *UAS-OPHN1-HA*.

For RNA interference experiments in BG2-c2 cells, *Graf* double-stranded RNA (dsRNA) was generated by in vitro transcription of a DNA template containing the T7 promoter sequence at both ends, as previously described [26]. The DNA template was PCR-amplified from *UAS-Graf-HA* using primers containing the T7 promoter sequence upstream of the following *Graf*-specific sequences: 5′-AATTTGAGTGCGATGAAGTTC-3′ and 5′- ATTTCAACATTCTACGTTTTC-3′. The efficiency of *Graf* knockdown was confirmed by RT-PCR analysis using the following primers: 5′-GCAAACGCAAACATCATGGGC-3′ and 5′-GAGTGGACAGGATCTTTGCCG-3′ for *Graf*, and 5′-CACCAGTCGGATCGATATGC-3′ and 5′-CACGTTGTGCACCAGGAACT-3′ for *rp49*.

### Brain dissection and immunostaining

Brains from pupae or 2-day-old adults were dissected in ice-cold phosphate-buffered saline (PBS) and fixed in PBS containing 4% formaldehyde for 30 min. Fixed brains were washed three times for 20 min each with PBS containing 0.3% Triton X-100, and blocked in PBS containing 0.3% Triton X-100 and 0.2% bovine serum albumin for 30 min. Samples were sequentially incubated with primary antibodies in blocking buffer for 48 h at 4 °C and with fluorescently labeled secondary antibodies overnight at 4 °C. The following primary antibodies were used: mouse anti-FasII (1D4, 1:10; Developmental Studies Hybridoma Bank, Iowa City, IA, USA), rabbit anti-phospho-ERK (1:500; Cell Signaling Technology, Danvers, MA, USA), and rabbit anti-GFP (1:1000; Invitrogen). FITC- and Cy3-conjugated secondary antibodies were purchased from Jackson ImmunoResearch (West Grove, PA, USA) and used at a dilution of 1:200. Images were captured with either an LSM 800 laser-scanning confocal microscope (Carl Zeiss, Jena, Germany) using a Plan Apo 20 × /0.8 objective lens or an FV300 laser-scanning confocal microscope (Olympus, Tokyo, Japan) using a Plan Apo 10 × /0.45 or Plan Apo 20 × /0.80 objective lens.

### Western blotting

Brains from 2-day-old adults were homogenized in sodium dodecyl sulfate (SDS) sample buffer (62.5 mM Tris–HCl, pH 6.8, 10% glycerol, 2% SDS, 2.88 mM β-mercaptoethanol, and 0.02% bromophenol blue). Samples were boiled for 5 min and centrifuged at 13,000 g for 10 min. Supernatants were subjected to 10% SDS-PAGE and then transferred to polyvinylidene fluoride membrane (Merck Millipore, Burlington, MA, USA). Blots were incubated overnight at 4 °C with rabbit anti-phospho-ERK (1:1000, Cell Signaling Technology) or rabbit anti-ERK (1:1000, Cell Signaling Technology), and then for 1 h at 25 °C with HRP-conjugated secondary antibody (1:5000; Jackson ImmunoResearch) in blocking solution (5% skim milk/0.1% Tween-20/Tris-buffered saline). Protein bands were detected using ECL reagents (iNtRON Biotechnology, Seongnam, Republic of Korea).

### Cell transfection

*Drosophila* neuronal BG2-c2 cells (DGRC, Bloomington, IN, USA) were maintained at 25 °C in Shields and Sang M3 insect medium (Sigma-Aldrich, St. Louis, MO, USA) supplemented with 10% heat-inactivated fetal bovine serum (Gibco, Carlsbad, CA, USA) and 10 μg/ml insulin (Sigma-Aldrich). *Drosophila* S2R + cells were maintained at 25 °C in Schneider’s medium (Gibco) supplemented with 10% heat-inactivated fetal bovine serum. Cells were transfected in serum-free medium using Cellfectin II (Invitrogen), according to the manufacturer’s instructions.

### EGFR internalization assay

BG2-c2 cells were transfected with *UAS-Flag-EGFR* and *actin-C5-GAL4* in the presence or absence of *Graf* dsRNA. At 48 h post-transfection, cells were starved for 6 h in serum-free medium and incubated with 5 μg/ml mouse anti-Flag antibody (Sigma-Aldrich) in serum-free medium for 1 h at 4 °C to label cell surface Flag-tagged EGFR proteins. After washing with serum-free medium, cells were incubated in Spitz (Spi)-conditioned medium, produced using S2R + cells as described in our previous study [19], containing 0 or 10 ng/ml Spi-HA for 5 min at 25 °C to allow for Flag-EGFR internalization. Cells were fixed in PBS containing 4% formaldehyde for 10 min and incubated with FITC-conjugated anti-mouse IgG secondary antibody (1:200 dilution) to label Flag-tagged EGFRs remaining on the cell surface. Cells were then washed and permeabilized with 0.2% Triton X-100 in PBS for 10 min and incubated with Cy3-conjugated anti-mouse IgG secondary antibody. A Z stack of optical sections (0.35 um thick) was taken with an LSM 800 laser-scanning confocal microscope (Carl Zeiss) using a Plan Apo 63 × /1.4 oil objective lens. ImageJ software (NIH, Frederick, MD, USA) was used to measure surface and internal Flag-EGFR fluorescence intensities as the integrated pixel intensities in the green and red channels, respectively. The internalization index was defined as the ratio of internalized to surface mean fluorescence intensities.

### Pavlovian olfactory learning and memory

Flies were maintained on a 12 h light/dark cycle at 25 °C. Four- to eight-day-old adult flies were subjected to a classical (Pavlovian) olfactory conditioning procedure under dim red light at 25 °C and 70% relative humidity, as previously described [27]. Briefly, 60 flies were collected in a training chamber, the inside of which was covered with a copper grid. Flies were allowed 30 s to acclimate and then sequentially exposed to two odors, 3-octanol (OCT; Sigma-Aldrich) and 4-methylcyclohexanol (MCH; Sigma-Aldrich). Relative concentrations of the two odors were adjusted so that naïve untrained flies had no preference for either of them in the T-maze (see below). Flies were first exposed for 60 s to the conditioned stimulus (CS + ; OCT or MCH) paired with the unconditioned stimulus (US; twelve 1.25 s pulses of 90 V electric shock delivered once every 5 s). After ventilation with fresh air for 30 s, flies were exposed for 60 s to the control stimulus (CS−; OCT or MCH) without electric shock. The chamber was then flushed with fresh air for 30 s. Each individual experiment consisted of two groups of 60 flies, with one group trained to OCT and the other group to MCH.

Trained flies were tested in a T-maze apparatus in which the OCT and MCH odors were simultaneously presented to the flies from the opposite arms of the maze. The flies located in the center of the maze were allowed to move freely for 2 min. Tested flies were trapped inside their respective T-maze arms, anesthetized, and counted. The performance index (PI) was calculated as the percentage of flies that correctly chose the CS− odor minus the percentage of flies that incorrectly chose the shock-associated CS + odor. A final PI was calculated by averaging both reciprocal PIs for the two odors.

Animals were subjected to one training session for learning, short-term (1 h) memory, and intermediate-term (3 h) memory experiments and to ten training sessions with a 15 min rest interval between each for long-term (24 h) memory experiments. For learning experiments, flies were immediately tested after training.

## Results

### ***Graf*** is required for normal MB β-lobe extension

**Fig. 1:**
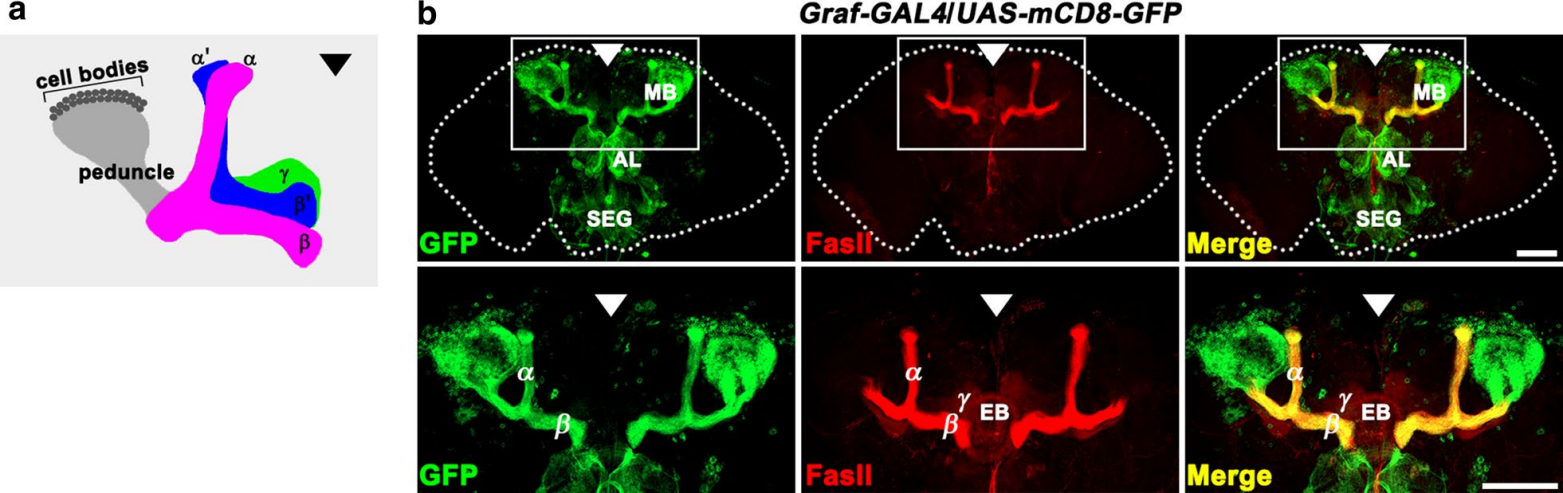
*Graf* promoter-driven expression of mCD8-GFP in adult MB neurons. **a** Schematic representation of the *Drosophila* adult MB (left side only) showing the axonal projections of α/β, α′/β′, and γ neurons. **b** Confocal z-projections of a wild-type adult brain expressing *UAS-mCD8-GFP* driven by a *Graf* promotor-*GAL4* fusion (*Graf-GAL4*) and stained with anti-GFP and anti-FasII antibodies. Bottom panels show high magnification views of areas marked by white boxes to highlight high expression of GFP in FasII-positive MB α/β axons. Note that the *Graf* promotor is additionally active in the antennal lobe (AL) and subesophageal ganglion (SEG). EB, ellipsoid body. Arrowheads indicate the brain midline. Scale bars, 100 μm

*Drosophila* MBs are bilaterally symmetrical neuropil structures with their cell bodies clustered in the dorsoposterior cortex of the fly brain. At the adult stage, each MB is composed of three major types of neurons: α/β, α′/β′, and γ [28]. These neurons project their axons ventroanteriorly through a tract called the peduncle (Fig. 1a). At the anterior extremity of the peduncle, individual axons from α/β and α′/β′ neurons bifurcate to form the dorsal (α and α′) and medial (β and β′) lobes. By contrast, γ neurons project only to the medial γ lobe. The lateral lobes (β, β′, and γ) in the adult MB terminate near the brain midline (Fig. 1a).

To investigate the roles of *Drosophila* Graf in MB development, we first explored its expression in the adult brain. As our polyclonal anti-Graf antibodies failed to detect endogenous Graf, we utilized *UAS-mCD8-GFP* (a UAS transgene of a membrane-associated mCD8-GFP) expression with a *Graf* promoter-*GAL4* fusion (*Graf-GAL4*). Prominent activity of *Graf-GAL4* was restricted in the MB, antennal lobe (AL), and subesophageal ganglion (SEG), as visualized by mCD8-GFP expression (Fig. 1b). Within the MB, *Graf-GAL4* activity was highly specific for α/β neurons (Fig. 1b).

**Fig. 2:**
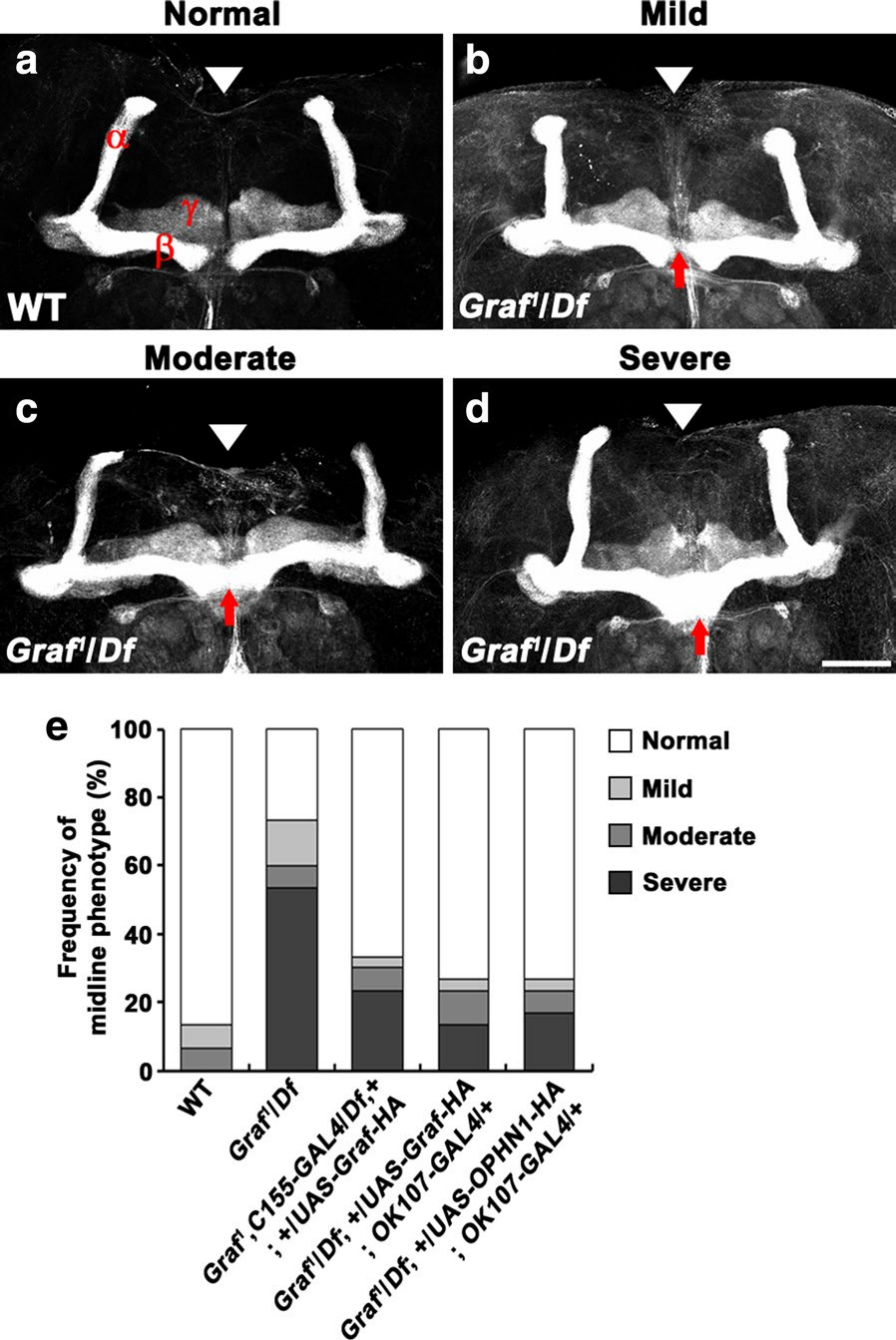
Mutation in *Graf* causes MB β lobes to over-extend beyond the brain midline. **a-d** Confocal z-projections of adult brains immunostained with anti-FasII antibody, which strongly labels the α and β lobes and weakly labels the γ lobe. **a** Wild-type (WT) brain with normal MB morphology. The lateral β and γ lobes from both hemispheres terminate near the brain midline without crossing it. **b-d** *Graf* mutant (*Graf*^*1*^/*Df*) brains exhibiting mild (**b**), moderate (**c**), and severe (**d**) levels of midline crossing by the β lobes (arrows). A mild phenotype was defined as a thin band of axon fibers crossing the midline; a moderate phenotype was defined as a substantial fiber bundle crossing the midline that was narrower than the width of the β-lobe termini; a severe phenotype was defined as a densely stained bundle crossing the midline that was equal to or greater in width than the adjacent β lobes [31]. **e** Quantification of the β-lobe midline-crossing phenotype in wild-type, *Graf*^*1*^/*Df*, *Graf*^*1*^,*C155-GAL4*/*Df*, + ; + /*UAS-Graf-HA*, *Graf*^*1*^/*Df*; + /*UAS-Graf-HA*; *OK107-GAL4*/ + , and *Graf*^*1*^/*Df*; + /*UAS-OPHN1-HA*; *OK107-GAL4*/ + adult flies (*n* = 30 brains). Arrowheads indicate the brain midline. Scale bar, 50 μm

We next assessed the overall morphology of the MBs from adult flies transheterozygous for the *Graf* null allele, *Graf*^*1*^ [19], and *Df(1)BSC756* (hereafter referred to as *Df*), a deficiency uncovering the *Graf* locus. For this, we used an antibody against the cell adhesion molecule fasciclin II (FasII) that strongly labels the α and β lobes strongly and weakly labels the γ lobe [29]. In wild-type (*w*^*1118*^) adult brains, axons in the medially projecting β and γ lobes terminated near the brain midline and rarely crossed it (Fig. 2a, e), as previously reported [30]. By contrast, in a majority (73%) of *Graf*^*1*^/*Df* mutant adult brains, β-axon fibers overextended beyond the midline, such that the β lobes from both hemispheres appeared to fuse (Fig. 2b–d). When quantified according to the scoring criteria described by Michel et al. [31], the β-lobe midline crossing phenotype of *Graf*^*1*^/*Df* mutant brains was categorized as mild (13%), moderate (7%), or severe (53%) (Fig. 2e). However, the projections of the α and γ lobes remained intact in *Graf*^*1*^/*Df* mutant brains (Fig. 2b–d), suggesting a β-lobe-specific role of *Graf*.

To test whether loss of *Graf* function is responsible for the observed β-lobe midline-crossing phenotype, we pursued rescue experiments by expressing hemagglutinin (HA)-tagged *Graf* (*UAS-Graf-HA*) with the pan-neuronal driver *C155-GAL4* or the pan-MB neuronal driver *OK107-GAL4* in the *Graf* mutant background. The transgenic expression of *Graf-HA* significantly reduced the severity of the β-lobe midline-crossing phenotype (Fig. 2e), demonstrating that *Graf* is required in MB neurons to correctly pattern β-lobe projections. We then examined whether MB-specific expression of human OPHN1 would rescue the β-lobe phenotype of *Graf* mutants. Expression of *UAS-OPHN1-HA* under the control of *OK107-GAL4* also significantly ameliorated the β-lobe midline-crossing phenotype of *Graf* mutants (Fig. 2e), indicating that human OPHN1 is a functional homolog of Graf.

**Fig. 3:**
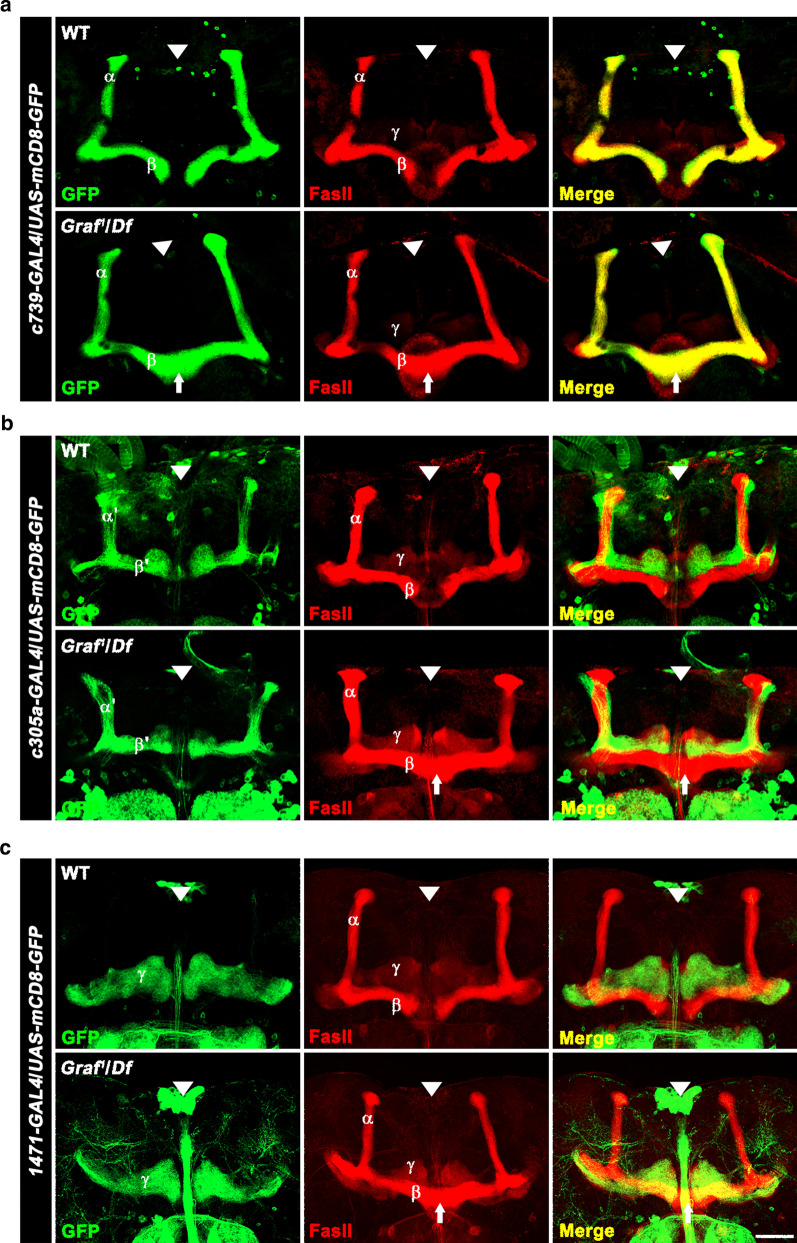
Loss of *Graf* selectively disrupts β-lobe morphology. Representative confocal z-projections of adult wild-type and *Graf*^*1*^/*Df* brains expressing *UAS-mCD8-GFP* under the control of MB cell-type specific GAL4 drivers to label all α/β (**a**; *c739*), α′/β′ (**b**; *c305a*), and γ (**c**; *1471*) neurons (*n* = 30 brains). Brains were double stained with anti-GFP (green) and anti-FasII (red) antibodies. Note that β lobes in *Graf* mutants display midline crossing defects (arrows), whereas the α, α′, β′, and γ lobes show no obvious morphological defect. Arrowheads indicate the brain midline. Scale bar, 50 μm

Because anti-FasII does not label the α′ and β′ lobes and only weakly stains the γ lobe, we also assessed the structure of wild-type and *Graf* mutant MBs by expressing *UAS-mCD8-GFP* using GAL4 drivers specific for MB α/β (*c739-GAL4*), α′/β ′ (*c305a-GAL4*), or γ (*1471-GAL4*) neurons [23]. In wild-type adult flies carrying *UAS-mCD8-GFP* and *c739-GAL4*, GFP-positive axons completely overlapped FasII immunostaining in the dorsal α and medial β lobes (Fig. 3a). *Graf*^*1*^/*Df* brains carrying *UAS-mCD8-GFP* and *c739-GAL4* showed GFP-positive β lobes crossing the midline, though their GFP-labeled *α* lobes were morphologically normal (Fig. 3a). The penetrance and expressivity of the β-lobe midline-crossing phenotype determined by GFP staining were not significantly different from those determined by anti-FasII immunohistochemistry. Importantly, the overall structures of the *UAS-mCD8-GFP*/*c305a-GAL4*-labeled α′ and β′ lobes and the *UAS-mCD8-GFP*/*1471-GAL4*-labeled γ lobes remained normal in *Graf*^*1*^/*Df* mutant brains (Fig. 3b, c), supporting the notion that the function of *Graf* in MB development is β lobe specific.

### The β-lobe midline-crossing phenotype of *Graf* mutant MBs develops during metamorphosis

**Fig. 4:**
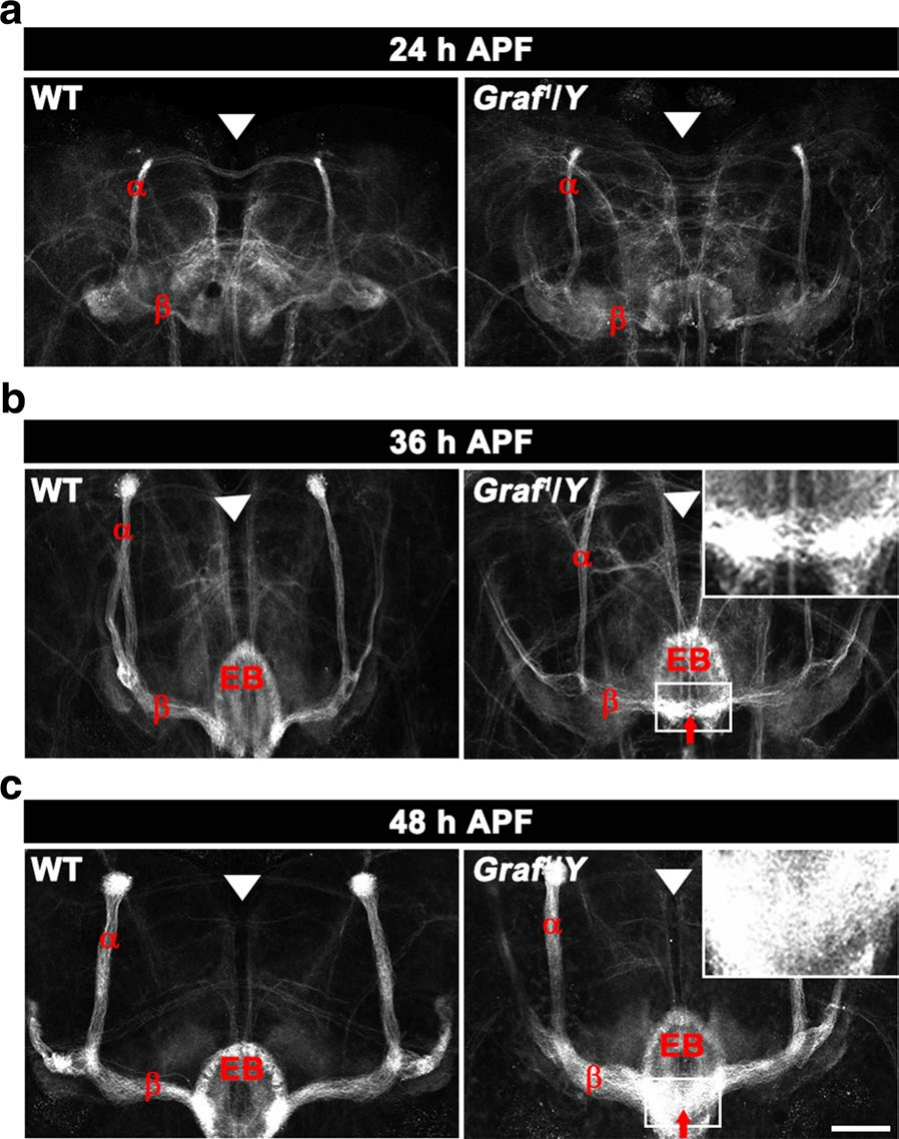
The β-lobe midline-crossing phenotype in *Graf* mutants develops during pupal development. Representative confocal z-projections of anti-FasII-labeled α and β lobes from wild-type and *Graf*^*1*^/*Df* pupae at indicated stages (*n* = 24 brains). **a** At 24 h APF, the medial β lobes extend toward the brain midline (arrowheads) in both wild-type and *Graf* mutant flies. At this developmental stage, the α and β lobes are very thin compared with their mature morphology. **b** At 36 h APF, β-lobe termini project onto the ellipsoid body (EB) without crossing the brain midline in the wild-type fly, whereas β-lobe fibers in the *Graf* mutant brain have crossed the midline (arrow). **c** At 48 h APF, MB lobes have thickened, and the β-lobe midline-crossing phenotype (arrow) can be seen more clearly. The insets show magnified regions of the midline. Scale bar, 50 μm

MB α/β neurons are generated and develop their axonal projections into the α and β lobes during the early pupal period [28]. To detect the onset of the β-lobe midline-crossing phenotype, we analyzed *Graf* mutant brains at early pupal stages via FasII immunostaining. In wild-type pupae at 24 h after puparium formation (APF), FasII labeling detected the nascent α and β lobes as thin dorsally and medially projecting bundles, respectively (Fig. 4a). At this developmental stage, the tips of the β lobes closely approached but did not cross the brain midline (Fig. 4a). The overall morphology of the α and β lobes was similar at 36 h APF and 48 h APF, except for an incremental increase of lobe thickness (Fig. 4b, c). In *Graf* mutants, the α and β lobes appeared as FasII-positive thin bundles with normal wild-type projections at 24 h APF (Fig. 4a), suggesting that axonal development of α/β neurons proceeded normally up to this stage. Furthermore, *Graf* mutant α and β lobes showed normal progressive thickening from 24 h APF onwards (Fig. 4b, c). However, the β-lobe midline-crossing phenotype was observed in *Graf* mutant brains at 36 h APF (19%; Fig. 4b) and was more pronounced at 48 h APF (30%; Fig. 4c), often leading to complete fusion of the two contralateral β lobes. These data indicate that the β-lobe midline-crossing phenotype of adult *Graf* mutants arises from an initial defect of axon extension during metamorphosis.

### Graf regulates β-lobe extension via a cell-autonomous mechanism

**Fig. 5:**
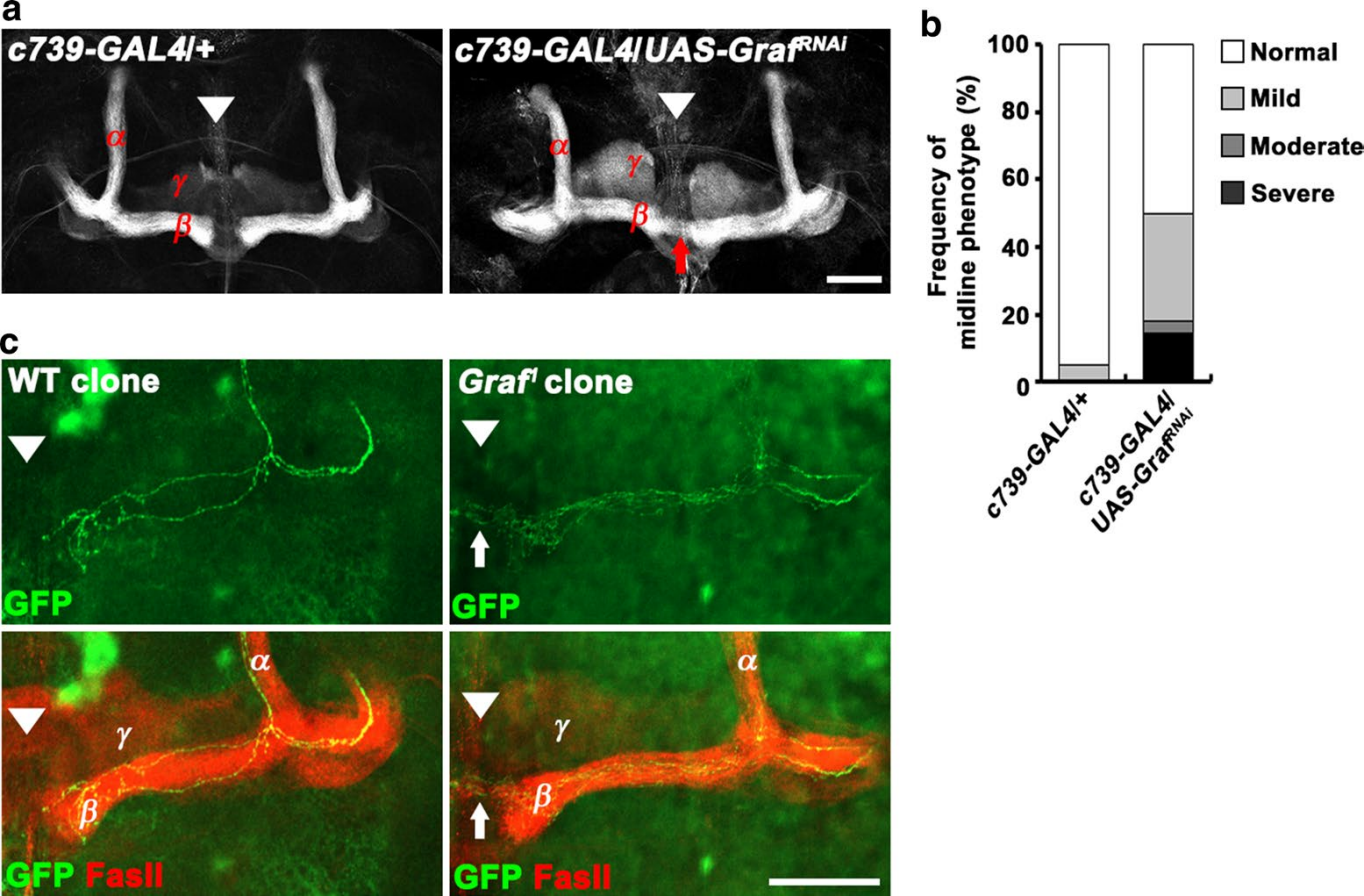
*Graf* is expressed in MB α/β neurons and acts cell autonomously to regulate β-lobe midline stopping. **a**, **b** Knocking down *Graf* expression specifically in MB α/β neurons induces midline crossing of β-lobe axons. **a** Confocal z-projections of *c739-GAL4*/ + and *c739-GAL4*/*UAS-Graf*^*RNAi*^ adult brains stained with anti-FasII antibody. **b** Quantification of the β-lobe midline-crossing phenotype. *c739-GAL4*/ + , *n* = 20 brains; *c739-GAL4*/*UAS-Graf*^*RNAi*^, *n* = 28 brains. **c** Confocal z-projections of wild-type and *Graf* mutant MARCM clones labeled with *UAS-mCD8-GFP* driven by *OK107-GAL4*. Brains were co-stained with anti-FasII to label MB α and β lobes. Genotypes: *hs-FLP*,*tubP-GAL80*,*FRT19A*/*FRT19A*; *UAS-mCD8-GFP*/ + ; *OK107-GAL4*/ + (wild-type clones) and *hs-FLP*,*tubP-GAL80*,*FRT19A*/*Graf*^*1*^,*FRT19A*; *UAS-mCD8-GFP*/ + ; *OK107-GAL4*/ + (*Graf*^*1*^ clones). Note that *Graf* mutant β axons in a *Graf*^*1*^*/* + heterozygous brain exceed the β-lobe region, further extending to the contralateral side. Arrowheads indicate the brain midline. Arrows indicate β-axons crossing the midline. Scale bars, 50 μm

To determine where Graf might act, we knocked down Graf specifically in MB α/β neurons. Expression of a *Graf* RNA-interference transgene (*Graf*^*RNAi*^) using the α/β neuron-specific *c739-GAL4* driver induced midline crossing of β axons in ~ 50% of the brains (Fig. 5a, b), supporting the possibility that Graf is required in α/β neurons for β-lobe midline stopping.

To further investigate cell autonomy, we generated single cell *Graf* mutant clones in the *Graf*^*1*^/ + heterozygous background using the MARCM technique [32], in which clones are produced by mitotic recombination and labeled by mCD8-GFP expression. In these experiments, we induced clone formation at the pupal stage to preferentially target α/β neurons. Similar to that observed for wild-type brains, a small minority of *Graf*^*1*^/ + heterozygous brains showed mild β-lobe midline-crossing phenotypes. We therefore quantified clonal axon phenotypes only in *Graf*^*1*^/ + brains with normal β-lobe morphology, revealing ~ 21% of *Graf*^*1*^ mutant clones exhibited a β-axon midline-crossing phenotype (Fig. 5c). In control experiments, none of the wild-type clones in the wild-type background displayed defects in β-axon extension (Fig. 5c). These findings support the model that Graf functions cell autonomously to prevent β-lobe overextension.

### Graf prevents β-lobe overextension by inhibiting EGFR signaling

**Fig. 6:**
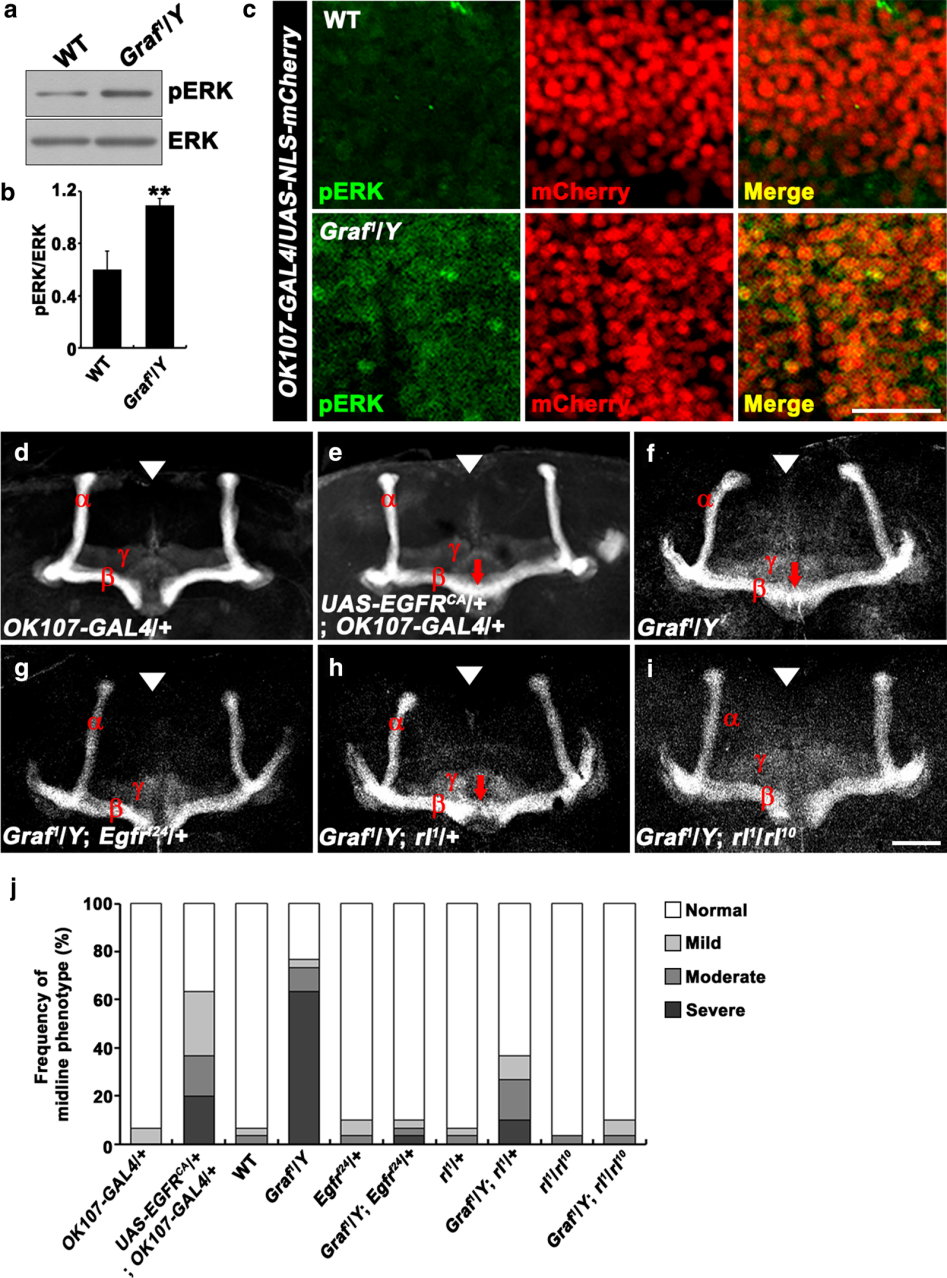
Genetic interactions for the β-lobe overextension defect between *Graf* and EGFR signaling pathway components. **a** Western blot analysis of brain lysates prepared from wild-type and *Graf*^*1*^/*Y* adult brains, using anti-pERK and anti-ERK antibodies. **b** Normalized ratios of pERK to ERK from three separate blots (**P < 0.01; Student’s *t* test). **c** Single confocal sections showing the pERK immunoreactivity in the MB neurons of *UAS-NLS-mCherry*/ + ; *OK107-GAL4*/ + (wild type) and *Graf*^*1*^/*Y*; *UAS-NLS-mCherry*/ + ; *OK107-GAL4*/ + (*Graf*^*1*^/*Y*) adult brains. Scale bar, 20 μm. **d**–**i** Sample confocal z-projections of anti-FasII-stained adult brains in *OK107-GAL4*/ + (**d**), *UAS-EGFR*^*CA*^/ + ; *OK107-GAL4*/ + (**e**), *Graf*^*1*^/*Y* (**e**), *Graf*^*1*^/*Y*; *Egfr*^*f24*^/ + (**g**), *Graf*^*1*^/*Y*; *rl*^*1*^/ + (**h**), and *Graf*^*1*^/*Y*; *rl*^*1*^/*rl*^*10*^ (**i**) flies. Arrowheads indicate the brain midline. Note that reduction or loss of EGFR signaling pathway components suppresses β-lobe overextension (arrows) in *Graf* mutant brains. Scale bar, 50 μm. **j** Quantification of the β-lobe midline-crossing phenotype in adult brains of indicated genotypes (*n* = 30 brains)

How does Graf regulate the extension of the tips of the MB β lobe? Graf plays an essential role in glycosylphosphatidylinositol-enriched endocytic compartment (GEEC) endocytosis [19]. In *Drosophila* blood cells (hemocytes), Graf-mediated GEEC endocytosis downregulates EGFR cell surface expression to negatively regulate EGFR-MAPK signaling [19], which plays a role in MB development [33]. We therefore hypothesized that Graf might regulate β-lobe development by way of EGFR signaling. To test this, we examined phosphorylated ERK (pERK), a readout of EGFR-MAPK activation [34], by western blot analysis of adult brain lysates. We observed a 1.8-fold-increase in pERK levels in hemizygous *Graf*^*1*^ brains relative to wild-type controls (Fig. 6a, b). Immunohistochemistry on adult brains also revealed increased pERK levels in *Graf* mutant MB neurons, which were labeled by *OK107-GAL4*/*UAS-NLS-mCherry* (Fig. 6c), confirming that Graf negatively regulates EGFR signaling in MB neurons.

We also examined the effect of constitutive activation of EGFR signaling on β-lobe extension. Overexpression of a UAS transgene of constitutively active EGFR (EGFR^CA^) using *OK107-GAL4* led to β-lobe overextension in the wild-type background, recapitulating the *Graf* null phenotype (Fig. 6d, e, j). This phenotypic similarity suggests that Graf acts through an EGFR signaling pathway to facilitate β-lobe midline stopping.

We then investigated whether reduction or loss of EGFR-MAPK pathway components suppresses the β-lobe defect of *Graf* mutants. Loss of one copy of *Egfr* or the MAPK gene *rolled* (*rl*), which had no effect on β-lobe extension in the wild-type background, significantly suppressed the β-lobe midline-crossing phenotype of *Graf*^*1*^ hemizygotes (Fig. 6f–h, j). Moreover, the β-lobe defect of *Graf* was further suppressed by removing both copies of *rl* (Fig. 6i, j), indicating that β-lobe midline crossing in *Graf* mutants depends on the level of EGFR signaling. Combined with the effect of EGFR^CA^ on β-lobe development, these findings are consistent with the model in which Graf prevents β-lobe overextension by downregulating EGFR-MAPK signaling.

**Fig. 7:**
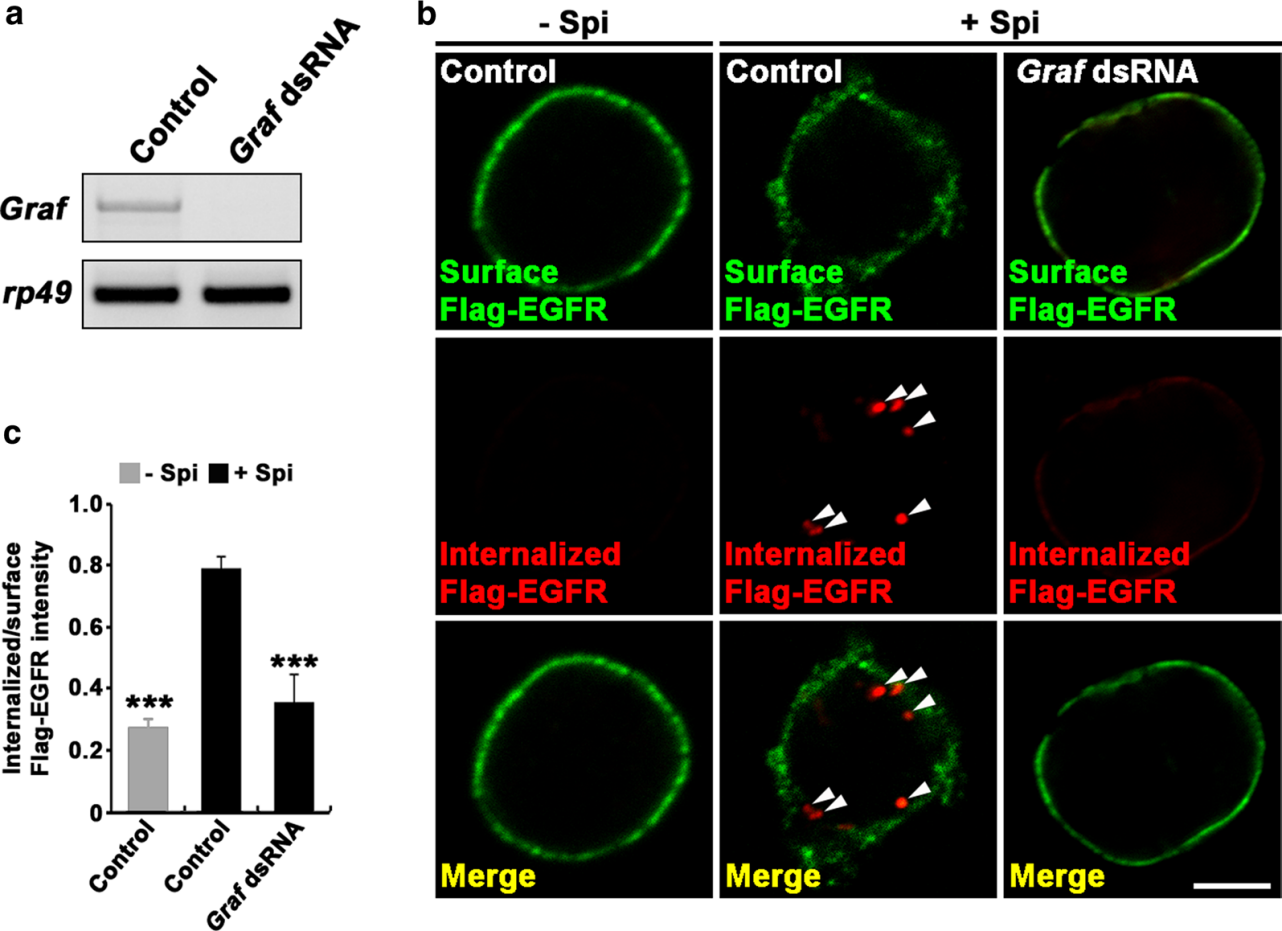
Graf is essential for ligand-induced EGFR internalization in BG2-c2 neuronal cells. **a** Quantitative RT-PCR analysis showing *Graf* RNA expression in control and *Graf*-knockdown BG2-c2 cells. *rp49* was a loading control. **b**, **c** Graf is required for Spi-induced internalization of EGFRs on the plasma membrane in BG2-c2 cells. Cells were transfected with a Flag-EGFR construct alone (control) or together with *Graf* dsRNA, and surface Flag-EGFR receptors were then prelabeled with anti-Flag antibody at 4 °C for 30 min. Cells were subsequently incubated with conditioned medium containing 0 or 10 ng/ml Spi-HA at 25 °C for 5 min for internalization of prelabeled cell surface Flag-EGFRs. After fixation of cells, surface (green) and internalized (red) Flag-EGFRs were sequentially labeled with secondary antibodies under nonpermeant and permeant conditions, respectively. **b** Single confocal sections through the middle of BG2-c2. Arrowheads indicate endosomal structures containing internalized EGFRs. **c** Quantification of internal-to-surface Flag-EGFR ratio in the control versus *Graf*-knockdown cells. Data are presented as means ± SEMs (*n* = 15 cells). Comparisons were made against Spi-treated control (***P < 0.001; one-way analysis of variance with Tukey–Kramer post hoc test). Scale bar, 5 μm

Next, we examined the impact of *Graf* knockdown on the endocytic removal of cell surface EGFRs in BG2-c2 neuronal cells. We transiently transfected a Flag-EGFR construct into control and *Graf*-knockdown cells (Fig. 7a), and assessed their ability to internalize cell surface Flag-EGFRs in a receptor internalization assay. In this assay, cell surface Flag-EGFRs in live cells were prelabeled with anti-Flag antibody at 4 °C in the absence or presence of HA-tagged secreted Spi (sSpi-HA), an EGFR ligand. Cells were then incubated at room temperature for 5 min to allow endocytosis to occur, and the cell surface and internalized pools of the prelabeled Flag-EGFRs were labeled sequentially with green- and red-fluorescent secondary antibodies under nonpermeant and permeant conditions. In unstimulated control cells, we observed a low background level of internalized Flag-EGFRs (Fig. 7b). However, Spi stimulation caused cell surface Flag-EGFRs to rapidly internalize into intracellular small punctae near the plasma membrane that may represent early endosomes (Fig. 7b). The ratio of internalized to surface Flag-EGFR levels was significantly increased in Spi-stimulated cells relative to that in unstimulated controls (Fig. 7c). Importantly, this Spi-induced EGFR internalization was completely abrogated by *Graf* knockdown (Fig. 7c). As Graf-dependent GEEC endocytosis is essential for EGFR degradation and signal attenuation [19], these results further support the model that Graf facilitates β-lobe midline stopping by downregulating EGFR signaling.

### Loss of Graf impairs olfactory long-term memory

**Fig. 8:**
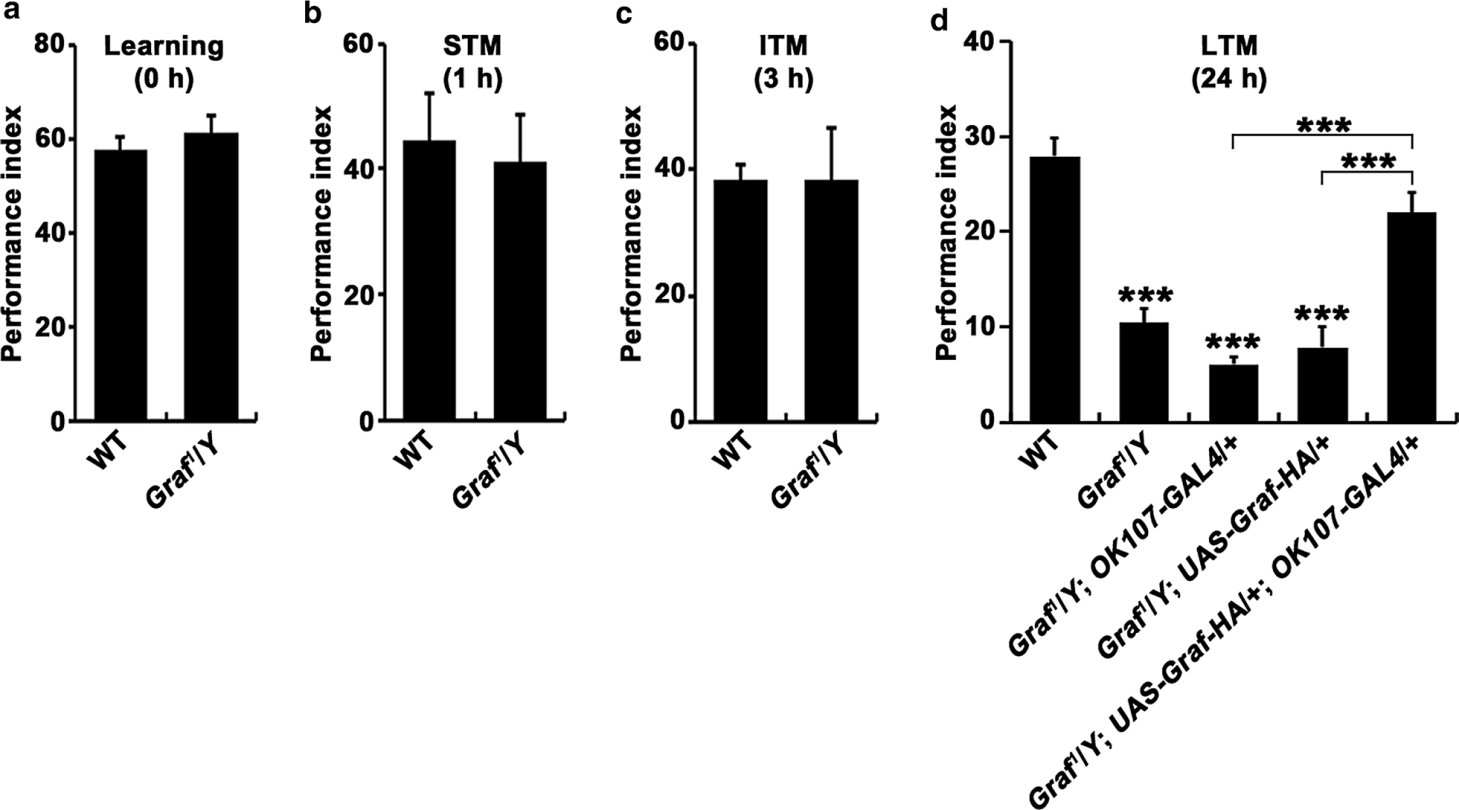
Long-term memory is defective in *Graf* mutants. Wild-type, *Graf*^*1*^/*Y*, *Graf*^*1*^/*Y*; *OK107-GAL4*/ + , *Graf*^*1*^/*Y*; *UAS-Graf-HA*/ + , and *Graf*^*1*^/*Y*; *UAS-Graf-HA*/ + ; *OK107-GAL4*/ + flies were tested for learning immediately after a single training session (**a**) as well as for short-term (1 h) (**b**), intermediate-term (3 h) (**c**), and long-term (24 h) (**d**) memory. Note that *Graf*^*1*^/*Y* mutants are specifically defective in long-term memory. Data are presented as means ± SEMs (*n* = 9 independent experiments). All comparisons are made against wild-type unless indicated (***P < 0.001; one-way analysis of variance with Tukey–Kramer post hoc test)

As the MB is a key center for learning and memory and cognitive impairment is a hallmark of ID, we tested the learning and memory abilities of hemizygous *Graf*^*1*^ mutants in an aversive olfactory learning assay [35]. In this assay, flies were trained to associate an electric shock with an air flow containing MCH or OCT. Neither wild-type nor *Graf* mutant flies showed a behavioral preference for either odor before training. Trained flies were then tested for their ability to remember the electric shock-associated odor in a T-maze, where both odors were delivered simultaneously in the absence of electric shock. Immediately after training (0 h), *Graf* mutant flies showed normal avoidance of the electric shock-associated odor, similarly to the wild-type flies (Fig. 8a), suggesting that olfactory learning is normal in *Graf* mutants. In addition, short-term (1 h) and intermediate-term (3 h) memories were also normally formed in *Graf* mutants (Fig. 8b, c). By contrast, *Graf* mutants displayed significantly reduced memory performance at 24 h after training (Fig. 8d). This phenotype of *Graf* mutants was significantly rescued by expressing *UAS-Graf-HA* using *OK107-GAL4* (Fig. 8d)*.* These data indicate that the loss of *Graf* selectively impairs long-term olfactory memory.

## Discussion

Here we revealed that *Drosophila* Graf is required for proper development of the MB, a primary brain center in the fly for olfactory learning and memory [36]. In normal MB development, the lateral β lobe terminates near the brain midline and rarely crosses it. However, axons of the β lobe in *Graf* mutants overextend beyond the midline, often resulting in apparent fusion of the two contralateral β lobes. Our evidence suggests that EGFR-MAPK signaling is involved in this *Graf* phenotype. First, levels of pERK are increased in *Graf* mutant MB neurons relative to wild-type controls, implying that Graf acts to downregulate EGFR-MAPK signaling. Second, MB neuron-specific expression of constitutively active EGFR also produces a β-lobe midline-crossing defect. Third, the midline-crossing phenotype of *Graf* mutants is suppressed by reducing the level of either *Egfr* or the MAPK gene *rl*. Finally, Graf mediates ligand-induced removal of cell surface EGFR in BG2-c2 neuronal cells. We previously showed that Graf-dependent internalization of EGFR is indispensable for its degradation and signal attenuation [19]. Thus, our present findings suggest that Graf downregulates EGFR-MAPK signaling to stop the β lobe from crossing the midline.

During MB development, the earlier-born α′/β′ and γ neurons establish lateral pathways that β axons can then follow [28, 37, 38]. However, the midline-crossing phenotype induced by *Graf* mutations or constitutive activation of EGFR signaling is restricted to the β lobe, with proper midline stopping of the β′ and γ lobes. This result suggests that the extension of β, β′, and γ lobes is independently controlled by lobe-specific mechanisms. Consistent with this idea, we found that the activity of the *Graf* promoter is highly specific for α/β neurons in the MB. Furthermore, a *Graf*-knockdown experiment and mosaic analysis demonstrated a cell-autonomous role for Graf in α/β neurons for proper stopping of the β lobe at the brain midline.

We also found that loss of Graf causes a specific defect in olfactory long-term memory, possibly paralleling cognitive impairments caused by OPHN1 loss in humans. It is generally accepted that the α/β neurons are necessary for olfactory long-term memory, whereas the α′/β′ and γ neurons mediate intermediate-and short-term memory, respectively [39–42]. Therefore, in *Graf* mutants, β-lobe midline crossing may account for the long-term memory deficit. Interestingly, simultaneous defects in β-lobe midline stopping and olfactory long-term memory are also caused by mutations in *dFmr1* [31, 43], which encodes an ortholog of the ID-associated protein FMRP [44]. This common feature of *Graf* and *dFmr1* supports the role of the β lobe in long-term memory and warrants future investigation of potential links between these two ID-associated genes.

Previous studies in mammals have implicated OPHN1 in the regulation of synaptic structure, function, and plasticity [9, 13, 14, 16, 17]. The present study uncovered a role for Graf in regulating axonal projections of memory-forming α/β neurons and provides a basis for further investigations into the neurodevelopmental roles of OPHN1 to gain additional insights into the pathogenesis of ID.

## Data Availability

Data sharing not applicable to this article as no datasets were generated or analysed during the current study.

## Abbreviations

APF: After puparium formation
EGFR: Epidermal growth factor receptor
Graf: GTPase regulator associated with focal adhesion kinase-1
GAP: GTPase-activating protein
ID: Intellectual disability
MAPK: Mitogen-activated protein kinase
MB: Mushroom body
MCH: 4-Methylcyclohexanol
OCT: 3-Octanol
OPHN1: Oligophrenin-1
Spi: Spitz
